# Stem Cells as Potential Candidates for Psoriasis Cell-Replacement Therapy

**DOI:** 10.3390/ijms18102182

**Published:** 2017-10-20

**Authors:** Agnieszka Owczarczyk-Saczonek, Magdalena Krajewska-Włodarczyk, Anna Kruszewska, Waldemar Placek, Wojciech Maksymowicz, Joanna Wojtkiewicz

**Affiliations:** 1 Department of Dermatology, Sexually Transmitted Diseases and Clinical Immunology, Faculty of Medicine, University of Warmia and Mazury in Olsztyn, 10-900 Olsztyn, Poland; alibra@o2.pl (A.K.); w.placek@wp.pl (W.P.); 2 Department of Rheumatology, Municipal Hospital in Olsztyn, 10-900 Olsztyn, Poland; magdalenakw@op.pl; 3 Department of Neurology and Neurosurgery, Faculty of Medicine, University of Warmia and Mazury in Olsztyn, 10-900 Olsztyn, Poland; maksymowicz@interia.pl; 4 Foundation for Nerve Cell Regeneration, University of Warmia and Mazury in Olsztyn, 10-900 Olsztyn, Poland; joanna.wojtkiewicz@uwm.edu.pl; 5 Department of Pathophysiology, Faculty of Medicine, University of Warmia and Mazury in Olsztyn, 10-900 Olsztyn, Poland; 6 Laboratory for Regenerative Medicine, Faculty of Medicine, University of Warmia and Mazury, 10-900 Olsztyn, Poland

**Keywords:** psoriasis, umbilical cord-Wharton’s Jelly stem cells, mesenchymal stem cells

## Abstract

Recent years have seen considerable progress in explaining the mechanisms of the pathogenesis of psoriasis, with a significant role played in it by the hyper-reactivity of Th1 and Th17 cells, Treg function disorder, as well as complex relationships between immune cells, keratinocytes, and vascular endothelium. The effect of stem cells in the epidermis and stem cells on T cells has been identified and the dysfunction of various types of stem cells may be a prime cause of dysregulation of the inflammatory response in psoriasis. However, exploring these mechanisms in detail could provide a chance to develop new therapeutic strategies. In this paper, the authors reviewed data on the role played by stem cells in the pathogenesis of psoriasis and initial attempts at using them in treatment.

## 1. Introduction

Psoriasis is a chronic disease affecting over 125 million people all over the world and an increasing trend has been observed [1,2]. As a systemic inflammatory process, it predisposes, twice as frequently than in the general population, to the development of metabolic disorders (insulin resistance, atherogenic dyslipidemia, arterial hypertension, and cardio-vascular diseases) and, consequently, to life shortening [3]. Moreover, this disease decreases the quality of life of the affected and leads to withdrawal from social life and development of depressive disorders. Patients with psoriasis present a significantly higher likelihood of suicidal thoughts, suicide attempts, and completed suicides [4]. Psoriasis constitutes a serious socioeconomic burden not only for patients but also for health care systems [5].

Unfortunately there is no fully satisfactory therapy against psoriasis and patients frequently report dissatisfaction with the treatment [6]. Although there has been some progress due to the introduction of biological therapies, it is still necessary to seek new therapeutic methods.

**Figure 1:**
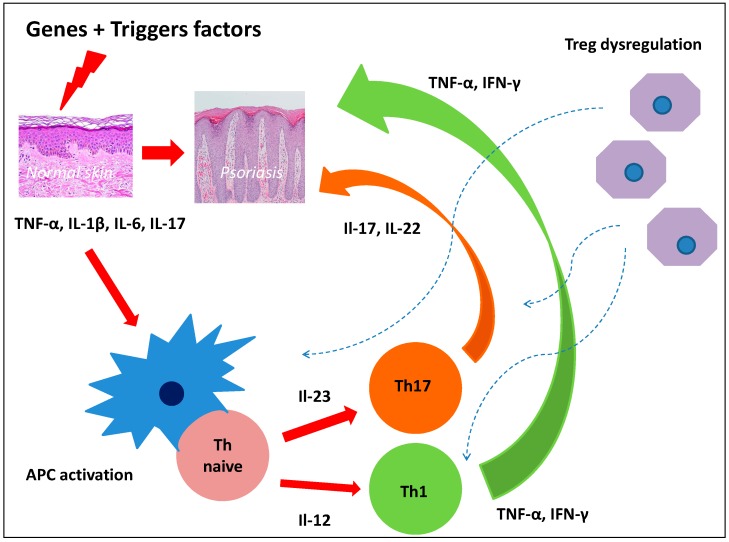
Dysregulation of the inflammatory response in psoriasis.

Although recent years have seen considerable progress in discovering the mechanisms of psoriasis pathogenesis, its full elucidation is still a long way away. Hyper-reactivity of Th1, Th17, dysregulation of Treg, and the complex relationships between immune system cells and keratinocytes and vascular endothelium obviously play a significant role [7]. Interleukin-23/Th17/Il-17 axis and Th1/IFN-γ axis play a key role in psoriasis inflammation [8]. Currently, attention is drawn to the effect of epidermal stem cells and stem cells on T cells. Therefore, the dysfunction of certain types of stem cells could be the root cause of dysregulation of the inflammatory response in psoriasis (Figure 1).

## 2. Pathogenesis of Psoriasis and Dermal Stem Cells

### 2.1. Epidermal Stem Cells in Psoriasis

**Figure 2:**
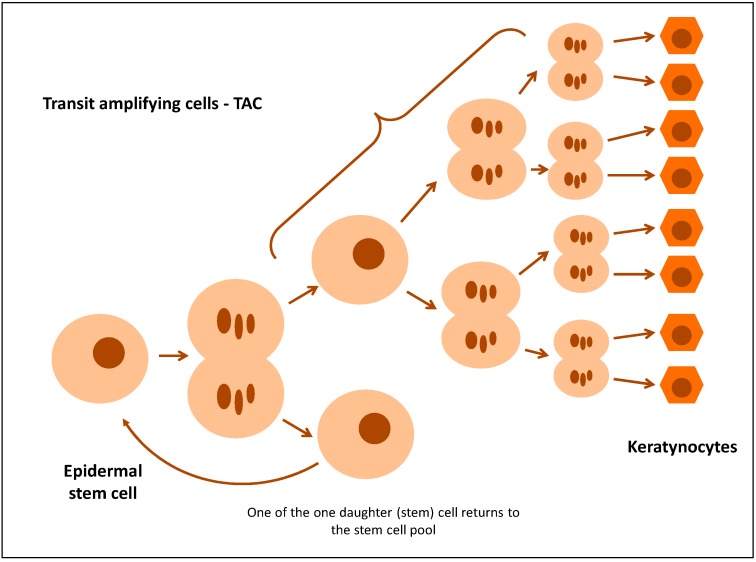
Regeneration of epidermis and the role of transit-amplifying cells.

Maintaining the right homeostasis in the epidermis requires constant recreation of the pool of keratinocytes. This is possible owing to the existence of stem cells (SC) and transit-amplifying cells (TAC) formed from them [9,10]. According to the classic stem cell division theory, it divides by asymmetric mitosis, yielding one daughter (stem) cell and one TAC. A TAC has limited potential for proliferation, as it divides a specific number of times before it is ultimately differentiated (Figure 2) [9,11,12]. TAC markers include nestin and E-FABP (Epidermal fatty acid-binding protein) and, to a lesser extent, β1-integrin, whose expression in normal epidermis is detected in the basal layer. It has been proposed that the integrin β1 expression level on the cell surface of basal keratinocytes is a key determinant which controls the transition of cells from the basal layer to the suprabasal layer [10,13]. Stem cells in which the level of expression of integrin β1 is higher than in TAC cells adhere strongly to the basal membrane, whereas TAC cells decrease the expression of integrin β1, which deprives them of adhesion capability. TAC cells with an integrin β1 deficit leave the basal layer and enter the suprabasal layer, while at the same time turning K15 transcription off and K10 transcription on [9,14]. This indicates that hyperproliferation of the epidermis in psoriasis is caused by an increase in the number of TAC cells [15]. 

An expanded suprabasal layer appears in psoriasis, which is caused by an increased population of transgenic progenitors [16]. According to Jia et al., excessive expansion of TAC cells is observed in the psoriatic epidermis, which exhausts the stem cell pool to the extent that K15-positive cells were barely detectable in the psoriatic basal layer [9]. The balance between the symmetric and asymmetric divisions of stem cells is the key to tissue homeostasis. Jia et al. suggest that a higher percentage of both symmetric and asymmetric cell division is observed in psoriasis, which is a mechanism responsible for acanthosis [9]. However, Charruyer et al. discovered that the number of symmetrical divisions increases in planoepithelial cancer and the number of asymmetrical divisions increases in psoriasis hyperproliferation (human and murine). Moreover, researchers discovered that IL-17A increases the number of asymmetrical divisions in human and murine keratinocytes [16]. On the other hand, Jia et al. proved that IL-17A and IL-22 inhibit differentiation of keratinocytes in order to maintain the stem cell phenotype and control the time of transition of cells from the basal layer to the suprabasal layer (an increase in the expression of K15 and integrin β1, markers of stem cells) [9].

E-FABP (interchangeably referred to as FABP5) is included in the FABP family of cytoplasmic proteins that bind long-chain fatty acids and other hydrophobic ligands. Their role also includes capture, transport, and metabolism of fatty acids. They participate not only in metabolic processes, but also act as signal molecules, thereby regulating inflammatory pathways [15,17]. Disorders of the E-FABP encoding gene were first identified in psoriasis. E-FABP regulates differentiation of keratinocytes, but the mechanism is not fully clear [18,19]. Moreover, it is known that E-FABP forms a complex with psoriasin (also known as S100A7). The protein also inhibits the activity of tyrosinase in normal melanocytes and melanoma cells, which reduces their proliferation [20]. Moreover, expression of E-FABP-naive T CD4^+^ cells promotes maturation of the Th17 line and inhibits Treg. This results in increased production of IL-17, which directly stimulates hyperproliferation of keratinocytes [18]. Blocking E-FABP may be a new therapeutic goal in psoriasis, preventing the development of metabolic syndrome in patients. 

Yamamoto et al. examined the activity of the stem cell factor (SCF), which is also a mast cell growth factor. An immunohistochemical examination demonstrated the presence of SCF in keratinocytes of psoriatic plaques, endothelium cells, and fibroblasts [21]. The serum levels of SCF were determined by ELISA. They were found to be elevated in patients with psoriasis compared to a healthy population. However, no correlation with the severity of the disease (PASI) was observed. SCF, released by keratinocytes, may cause the number of mast-cells in psoriatic eruptions to increase and induce pruritus within the eruptions [21].

### 2.2. Mesenchymal Stem Cells (MSC) in Psoriasis

Mesenchymal stem cells (MSC) in psoriatic plaques have an abnormal profile of cytokines that they secrete, which can influence the keratinocytes stimulating their proliferation and limiting their capability of apoptosis [22]. Liu et al. confirmed the influence of MSC from lesions on proliferation and apoptosis of HaCaT-cells (aneuploid immortal keratinocyte cell line from adult human skin). MSC exhibited similar expression of CD29, CD44, CD73, CD90, and CD105 to that in healthy skin, but with limited expression of CD34, CD45, and HLA-DR. This may explain their role in stimulation of the proliferation of psoriatic keratinocytes, which leads to acanthosis [22].

On the other hand, Orciani et al. assessed the basic functions of mesenchymal stem cells of skin, isolated from psoriatic patients: expression of inducible nitric oxide synthase (iNOS) and vascular endothelial growth factor (VEGF). They then compared them with the cells isolated from the skin of healthy people. The content of VEGF was the highest in MSC from the areas surrounding the psoriatic eruptions and that of iNOS—from lesions [23]. The secretion of these factors normalised after treatment with TNF-α inhibitors (etanercept, adalimumab) [24].

Similar conclusions were published by Hou et al. who found that the expression of genes associated with inflammation and angiogenesis, i.e., LITAF (lipopolysaccharide-induced tumor necrosis factor-alpha transcription factor), DUSP1 (dual-specificity protein phosphatase 1), VEGFα (vascular endothelial growth factor α), and IGFBP5 (insulin-like growth factor-binding protein-5) is increased in mesenchymal stem cells of the skin in psoriatic patients [25].

Recently, the authors compared the expression profile of miRNA in skin MSC in psoriatic patients and in healthy people by means of a micromatrix. Expression of pro-inflammatory miRNA miR-155 was found to be strongly increased in MCS in psoriatic patients [26]. As reported earlier by Xu et al., expression of mir-155 in psoriasis can be induced by proinflammatory cytokines, i.e., IFN-γ, TNF-α, or IL-1 [27]. Expression of mi-155 in MSC, DC cells, macrophages, and NK cells results in impaired immunosuppression, apoptosis of DC and an increase in the number of proinflammatory cytokines [28,29,30].

Additionally, miR-155 indirectly inhibits iNOS by inhibiting its target gene TAB2 [27]. This study showed that an increased level of mir-155 and decreased expression of iNOS in MSC in psoriatic patients can affect expression of cytokines and functions of immunoregulation in MSC. It was found that although expression of TAB2 and iNOS was low in MCS in psoriatic patients, expression of TGF-β and IL-10 (factors important for the function of MCS) was not significantly different in both groups, which suggests that MSC dysfunction in psoriasis is not induced by IGF-β and LI-10, but by mir-155 and iNOS [27].

MSC have immunosuppressive properties; they inhibit proliferation of activated T and B cells and cytotoxic activity of NK cells. Moreover, they change the profile of cytokines and chemokines secreted by dendritic cells and macrophages [31,32]. Psoriatic MSC have a decreased immunosuppressive capability. Liu et al. found a decreased ability to inhibit cell proliferation in psoriatic MSC co-cultured with activated T-cells in vitro, even after stimulation with IFN-γ together with TNF-α or IL-1β [32,33]. Moreover, a decreased antioxidative capability in psoriatic MSC was observed by Sah et al. They observed a decreased capability of psoriatic MSC using extracellular superoxide dismutase in eruptions induced by imiquimod [34].

MSC can differentiate into vascular endothelium cells. Excessive proliferation of capillary veins in psoriatic eruptions is observed earlier than in fully formed psoriatic plaque and it is responsible for Auspitz’s sign [35,36]. Niu et al. analyzed the expression of genes (measurement of mRNA) that induce angiogenesis in mesenchymal stem cells from the skin of psoriatic patients for PECAM 1 (platelet endothelial cell adhesion molecule-1), FGD5 (faciogenital dysplasia-5), PTGS1 (prostaglandin-endoperoxide synthase-1), MCAM (melanoma cell adhesion molecule), VASH2 (vasohibin-2), and STAB1 (stabilin-1). Considerably decreased expansion of PECAM1, PTGS1, FGD5, and MCAM in MCS was found, with no difference observed in the expression of VASH2 and STAB1 compared to healthy people. According to the authors, low expression of PECAM1 and MCAM may be interpreted as a method of inflammation modulation by dental matrix components (DMCs). However, their ability to differentiate in vitro into adipocytes and osteoblasts is preserved [37].

### 2.3. Role of Telocytes in Psoriasis

Human skin contains telocytes, which are parts of niches for stem cells interact with them closely, taking part in skin regeneration [38]. These are cells which have long, very thin projections called telopods, whose thickness is comparable to that of collagen fibers [39]. Manole et al. detected a reduced number of telocytes in psoriasis plaques and their degradation involves membrane disintegration, fragmentation of cytoplasm, and loss of the cell nuclei. On the other hand, topical treatment with corticosteroids increased their number [39].

## 3. Psoriasis and Bone Marrow Stem Cells

Although the role of T-cells in psoriasis has been very well elucidated, the cause of their hyper-reactivity remains unknown. Moreover, disorders in the function of other immune cells (monocytes, neutrophils, mast cells) are observed in psoriatic patients [40]. 

### 3.1. Associations of Psoriasis with Bone Marrow Cells

Although various factors upset the balance of the immune system in initiation of psoriatic lesions, the most important of them are associated with T cells (spontaneous activation and proliferation, production of proinflammatory cytokines). It has been suggested that hematopoietic stem cells (HSC) of bone marrow may be responsible for dysregulation of the T cells function in psoriasis. It has been shown in several recent studies that abnormal relations between T cells and HSC may be genetically conditioned. This has been proven by reported cases of remission of psoriasis, or vice-versa—the development of diseases after an allogeneic bone marrow transplant [40,41,42,43,44]. Immunological disorders typical of psoriasis can be transmitted through a bone marrow cell transplant. Leukemia in psoriatic patients can also result in the disease remission [44].

### 3.2. Proliferation of Bone Marrow Stem Cells (Progenitor Cells) in Psoriatic Patients Is Not Normal

In vitro studies with 99mTc bone marrow scintigraphy showed that the activity of monocytopoiesis precursors is increased in psoriasis. In consequence, the number of phagocytic cells in patients’ blood increases. Moreover, peripheral immunocytes and cytokines secreted by them (IFN-γ, IL-2, IL-8, and TNF-α) can affect the hematopoietic environment and even hematopoiesis [40,44].

Zhang et al. demonstrated that the activity of HPP-CFC (high proliferative potential colony-forming cell) and the ability to produce CFU-GM (granulocyte-macrophage colony-forming units), but not CFU-E (Erythrocyte colony-forming units), is decreased in psoriatic patients [44].

### 3.3. T-Cells from Haematopoietic Cells of Bone Marrow of Psoriatic Patients Are Functionally Different from T-Cells in Healthy People

Zhang et al. observed a culture of CD34^+^ cells of bone marrow from psoriatic patients and the process of their differentiation into T cells and regulatory CD25^+^ CD4^+^ cells. Studies have shown that CD4^+^ CD25^+^ cells inhibit effector T cells to a lesser extent both in peripheral blood and in skin lesions, which leads to their accelerated proliferation. Although the proportions of CD4^+^ CD25^+^ cells and expression of the FOXP3 gene are comparable in psoriatic patients and in healthy people, their proliferation and secretion of cytokines IL-2 and IL-10 is decreased in patients in response to the streptococcal antigen (Strep-A). Additionally, CD4^+^ CD25^+^ cells in patients cannot inhibit effector T cells [40,44].

### 3.4. The Role of Bone Marrow Mesenchymal Stem Cells in Psoriatic Patients

Bone marrow mesenchymal stem cells (BMSCs), also called bone marrow stromal cells, exhibit a pleiotropic immunomodulating effect, e.g., by participating in inhibition of T and NK cell proliferation and in maturation of dendritic cells [25,36]. Cytokines secreted by BMSC affect hematopoiesis. This is a group comprising over 30 hematopoietic growth factors and pro-inflammatory cytokines, including TNF-α, IL-1, IL-6, IL-7, IL-8, IL-10, IL-12, IFN-γ, and IL-18, which also affect immune response in peripheral blood. Secretion of granulocyte colony stimulation factor (G-CSF) and IL-6 increases in cultured BMSCs from psoriatic patients, whereas the levels of IL-1α, IL-1β, IL-3, IL-8, epidermal growth factor (EGF), vascular endothelial growth factor (VEGF), TNF-α, leukaemia inhibitory factor (LIF), hepatocyte growth factor (HGF), and platelet-derived growth factor (PDGF) is decreased and that of GM-CSF, IL-11, and IL-7 is unchanged. However, the concentrations of these cytokines are not correlated with PASI, which shows that their abnormal secretion results from an anomaly of BMSCs rather than from a systemic inflammatory response [25,36,44].

Mesenchymal stem cells isolated from psoriatic eruptions inhibit proliferation of T-cells less strongly than those from healthy people. Moreover, they exhibited increased secretion of IL-11, decreased secretion of IL-6 and HGF, while the production of TGF-β1 was unchanged [45].

BMSCs can differentiate into multiple mesenchymal lines, including osteocytes, chondrocytes, adipose cells, endothelial cells, and cells of skeletal muscles. Studies have shown that BMSCs taken from psoriatic patients are less able to proliferate and differentiate into these types, but they are more able to differentiate into vascular endothelial cells compared to those taken from healthy people. What is more, this ability is associated with the severity of the disease (PASI) [44].

Campanati et al. evaluated the expression of 43 genes encoding cytokines, typical of Th1, Th2, and Th17 in mesenchymal stem cells isolated from psoriatic eruptions. It was shown that expression of genes for cytokines of the Th1 and Th17 profile (INF-γ, CCR5, CXCL9, CXCL10, IL6, IL8, TNF-a, IL23A, CCL2, CCL20, CXCL2, CXCL5, IL17C, IL17F, IL17RA, IL21, TLR2) is higher in healthy people and it was similar in both groups for the Th2 profile (CCL1, CCL22, CXCL12, IL2, IL3, IL4, IL13B, IL22, IL27, TGF-β1) [24].

Furthermore, Hou et al. demonstrated disorders in the methylation of receptor genes of MSC, involved in signaling pathways [25].

### 3.5. Reduction of the Number of Progenitor Cells in Psoriatic Patients

Being a systemic inflammatory process, psoriasis leads to the development of metabolic disorders. By exhausting the pool of endothelial progenitor cells (EPCs) and being a source of pro-inflammatory cytokines, psoriatic eruptions that stay on the skin for years affect the cardiovascular system. It is a population of bone marrow cells with expression of CD34 and VGEFR-2 cells [45]. EPCs are responsible for integrity and regeneration of endothelium and the formation of new vessels in adults. They are recruited from bone marrow and migrate to areas of ischemia or damaged endothelium, where they repair the damage. Their number is reduced in arterial hypertension, diabetes, obesity, rheumatoid arthritis, and in tobacco smokers. Their number in blood is also reduced in patients with psoriasis and in those with psoriatic arthritis. However, the number of EPCs was found to increase following treatment with etanercept (TNF-α inhibitor), which is indicative of an improvement of the regenerative abilities of endothelium [46,47,48,49]. On the other hand, Ablin et al. did not find any significant differences between the number of EPCs in healthy people and those with psoriasis and psoriatic arthritis [45].

## 4. Stem Cells in Psoriasis Treatment

### 4.1. Autologous Haematopoietic Stem Cell Transplantation

Currently, the first clinical trials are being conducted into the use of stem cells in the treatment of psoriasis. This idea originated from observation of the remission of lesions in patients treated with mesenchymal or hematopoietic stem cells because of lymphomas and leukemias as well as other autoimmune diseases (diabetes, sclerosis multiplex, rheumatoid arthritis, systemic lupus erythematosus) [41,42,43,50] (Table 1). More than 30 patients with psoriasis who had bone marrow transplantation (BMT) which resulted in prolonged remission of psoriasis have been described in the literature in the past 25 years [42,51]. However, there have also been a few who acquired psoriasis after allogeneic BMT and blood transfusion from donors with this disease [42].

According to the pathogenesis of psoriasis, T-cells can provoke the development of changes after a blood transfusion, but peripheral T-cells have a relatively short life time. Psoriasis patients are known to enter remission after allogeneic transplants (but not after autologous transplants) of hematopoietic stem cells; this indicates that hematopoietic stem cells are the major factors provoking the disease [42,44].

Adkins et al. described the case of a 55-year-old female patient with chronic myeloid leukemia and concurrent refractory psoriasis, who achieved complete remission after an allogeneic bone marrow transplant. Admittedly, such quick remission may have been caused by medicines preparing the patient for the transplant (busulfan, cyclophosphamide) [41]. 

Likewise, Mori et al. described the case of a 54-year-old male patient with a 10-year history of psoriasis, who was treated by an allogeneic bone marrow transplant with preceding myeloablation with busulfan and cyclophosphamide because of a myelodysplastic syndrome, who achieved complete remission of psoriasis, which remained throughout the 8-month follow-up. The authors emphasize the role of the elimination of autoreactive lymphocytes during an allo-BMT, as well as immunosuppressive treatment in maintaining the state of remission [75].

Braiteh et al. presented the case of a 35-year-old patient with psoriasis (BSA 50%) and psoriatic arthritis (PsA), who had an autologous BMT (preceded by myeloablation with mephalen) in the treatment of multiple myeloma. A one-year remission of myeloma was achieved. No symptoms of psoriasis or PsA were observed during the follow-up program (more than two years). The authors emphasize that spectacular remissions were observed in patients with concurrent autoimmune diseases, who had auto hematopoietic stem cell transplantation (HSCT), but the mechanism of the effect remains unclear. Other studies have suggested that it is related to the “resetting” of the immune memory, which may result from the myeloablation preceding auto HSCT. It eliminates reactive lymphocytes and reduces reactive B cells and, in consequence, reduces auto-antibodies and developing immune tolerance. Repeated autologous infusion of stem cells may promote the regeneration of a population of lymphocytes which are able to distinguish the body’s own cells. Renewal of the population of regulatory T-cells and reactive B cells from naive progenitor cells exposed to the body’s own cells can be responsible for auto-tolerance. Moreover, auto HSCT can increase the production of cytokines and play a role in the regeneration of tissues damaged by inflammation [76]. 

Zurita et al. presented the results of a study of patients with severe psoriasis treated by an autologous transplant of haematopoietic cells (*n* = 30), compared with PUVA therapy (*n* = 19). Taking bone marrow from both iliac crests and isolation of the CD34^+^ fraction was followed by a single intravenous administration of autologous cells. The therapeutic effects were controlled for up to six months and compared with the effects of PUVA. PASI 75 reached a statistically significant level in the group treated with stem cells, but no significant difference was observed compared to the effects of PUVA [50].

### 4.2. Umbilical Cord-Wharton’s Jelly Stem Cells

Umbilical cord-Wharton’s Jelly stem cells (WJSCs) seem to be an ideal candidate for this therapy (Table 2). WJSCs are plastic-adherent when maintained in standard culture conditions. They express CD105, CD73, and CD90, as well as more recently recognized markers such as CD44, CD146, and CD166. However, they do not express CD3, CD45, CD34, CD14 or CD11b, CD45, CD144, CD79α or CD19, vascular endothelial growth factor (VEGF)-R1, VEGF-R2, and HLA-DR surface molecules [77,78]. Some UCB-derived cell populations show inherent ‘immunoprivileged’ properties because they exhibit class I HLA antigens, and class II HLA antigens are seen only in response to INF-γ [79]. These features fulfil the stipulated minimum criteria of ‘plastic adherence’, ‘immunological profile’, and ‘differentiation’ as stated in the position paper of the International Society for Cellular Therapy [77]. MSCs from within WJSCs are a relatively young cell type compared to most other MSCs. Among the many sources of stem cells, the human umbilical cord matrix, i.e., Wharton’s jelly (WJ), has recently become a preferential source of stem cells, because of its rapid availability with a large donor pool, non-invasive and painless collection, no risk for the donor, no ethical constraints, hypo-immunogenic and non-tumorigenic, high in vitro expandable rates and multi-potent differentiation potential, which makes them important sources for the isolation and banking of stem cells [80,81,82]. In addition, since they are rarely exposed to infectious agents, they represent a safe donor [83]. Chen et al. reported good results for psoriasis treatment using WJSCs in two cases. In the first, a patient (a 35-old-man with psoriasis and diffuse large B-cell lymphoma, stage IV) after hematopoietic stem cell transplantation failure, was successfully WJSCs-treated, with no recurrence of lymphoma or psoriasis. in the second patient, (a 26-year-old woman with psoriasis vulgaris), after three infusions, 1 × 10^6^/kg each time over three successive weeks and two more three months later) a complete remission of the disease was observed [84]. No recurrence of the disease was observed during the 4-year follow-up [84]. Similar effects were achieved in the treatment of psoriatic arthritis [43]. 

Human Wharton’s Jelly-derived Mesenchymal Stem Cells (hWJ-MSCs) display immunosuppressive properties and may be able to play an important role in autoimmune disorders [61]. Regulatory T-cells (Treg) are important in preventing autoimmune disease development. Interleukin-35 induces the proliferation of Treg cell populations and reduces the activity of Th17 and Th1 cells, which play a central role in the initiation of inflammation and autoimmune diseases. Recent studies identified IL-35 as a new inhibitory cytokine required for the suppressive function of Treg cells. Amari et al. revealed hWJ-MSCs as a good source of IL-35 for reduction of inflammation and autoimmune diseases [61].

Th17 cell homeostasis is the relationship with Tregs, whose imbalance may lead to the development of psoriasis. Rafei et al. confirmed that MSCs inhibit the activity of the Thl7 cell, reducing the expression of interleukin IL-17 and decreasing inflammatory cell infiltration in the central nervous system [68]. Park et al. found that TGF-β-transduced MSCs in experimental autoimmune arthritis suppressed T-cell proliferation, and down-regulated pro-inflammatory cytokine production. Moreover, these therapeutic effects were associated with an increase in CD4+FoxP3+ Treg cells, inhibition of Th17 cell formation, and inhibited osteoclast differentiation [89]. Alluno et al. revealed that only IFN-γ-pretreated microencapsulated (CpS)-hUCMS (not free) have properties to suppress T cell proliferation and restore the Treg/Th17 ratio in Sjögren syndrome [57]. They reciprocally modulate FoxP3 and RORγt expression, eventually leading to the conversion of Th17 into Treg cells, despite the presence of hUCMS-derived IL-6. Indeed, CD4+IL-17+FoxP3+RORγt− T-cells may represent an intermediate phenotype of Th17 cells turning into Treg cells [57]. Moreover, mesenchymal stem cells can up-regulate Treg transcription factor FoxP3 and Th17/Treg plasticity is well established [90]. Wang et al. assessed the effect of WJ-MSCs on Th1, Th2, Th17 cytokines production and Treg augmentation. WJ-MSCs co-cultured with peripheral blood mononuclear, showed an immunosuppressive function by inhibiting the proliferative response of Th1 and Th17 but augmenting Th2 and Treg. Moreover, co-cultures with WJ-MSCs resulted in an increase in IL-10 and IL-6 levels, especially in co-cultures with IFN-γ treated WJ-MSCs [58]. Additionally, they can affect maturation and activation of precursors of dendritic cells (DC) [56]. hWJSCs express CD14, a common marker for marcrophages. The soluble form of CD14 can down-regulate T cell activation [56,91].

WJSCs produce large amounts of tolerogenic IL-10, higher levels of TGF-β than BM-MSCs, and express HLA-G, which is not expressed in BM-MSCs [56,57]. Chen et al. detected therapeutic effects and the mechanism of WJ-MSC in a spontaneous-abortion rat model. The mechanism may be related to its up-regulation of IL-10 and down-regulation of IFN-γ and IL-17 [55].

hWJSCs had an up-regulation of the induction of apoptosis [64]. The increase in tumor cell death driven by WJ-MSCs such as Bcl-2, Bcl-xL, Survivin, Mcl-1, and cIAP-1 have been observed [74]. By cleaved caspase 3/9 up-regulation in cancer cells, WJ-MSC executed its pro-apoptotic effect [92,93].

hWJSCs express HLA-G, which is important for immune tolerance [64,65]. HLA-G is a non-classical major histo-compatibility complex (MHC) class I protein which is expressed in both membrane-bound and soluble isoforms that can display tolerogenic properties via interaction with inhibitory receptors on dendritic cells, natural killer cells, and T cells. Soluble HLA-G exerts an immunosuppressive effect by inducing apoptosis in CD8^+^ T cells, while also down-modulating CD4^+^ T cell proliferation. Unfortunately, the expressional level of HLA-G is higher in adult tissue compared to foetal tissue MSC [58,94]. 

## 5. Conclusions

The data presented here suggest that stem cells in psoriatic patients affect proliferation and differentiation of keratinocytes and immune system cells and the ability to secrete cytokines. This confirms the view that psoriasis is a multi-system disease, which affects not only the skin, but is also related to the function of the hematopoietic and neuroendocrine systems and with metabolic disorders. Although it requires a lot of new research (Table 3). The application of stem cells raises hopes for developing a new, safe, and effective therapy for psoriatic patients.

## Figures and Tables

**Table 1:** Analysis of the potential effect of stem cells on inhibition of the inflammatory process in psoriasis.

Element of Psoriasis Pathogenesis	Effect of Stem Cells	References
Deficiency of IL-10 and TGF-β in serum and skin [52,53,54]	WJSCs produce IL-10 and TGF-β	Chen et al., 2016 [55] Kim et al., 2013 [56] Prasanna et al., 2010 [57] Wang et al., 2016 [58]
Hyperactivity of Th17 and the dysfunction of Treg [59,60]	WJSCs produce IL-35, which induces the proliferation of Treg cell populations, reduces the activity of Th17 and Th1 cells	Amari et al., 2014 [61]
Significantly lower plasma levels of sHLA-G and IL-10 in psoriatic patients; the treatment of psoriasis leads to suppression of Th1 activation because of induced sHLA-G secretion via an IL-10-dependent pathway [62,63]	WJSCs express of HLA-G	Kim et al., 2013 [56] Prasanna et al., 2010 [57] Fong et al., 2011 [64] La Rocca et al., 2009 [65]
A crucial role of Th17 and IL-17 in psoriatic plaques and general inflammation [66,67]	MSCs inhibit the activity of the Thl7 cell, reducing the expression of interleukin IL-17	Rafei et al., 2009 [68]
Decrease in FOXP3 and an increase in IL-17-producing Tregs; the conversion from Treg to IL-17/Treg is a continuum of converting cells, as evidenced by FOXP3+ RORγt+ co-expression and a gradual loss of FOXP3 [69]	WJSCs modulate FoxP3 and RORγt expression, leading to the conversion of Th17 into Treg cells	Alluno et al., 2014 [70]
Excessively aberrant Th1/Th2 homeostasis and Th17/Treg balance; the dysfunction of Treg [7,17,71,72]	WJ-MSCs inhibit the proliferative response of Th1 and Th17 but augment Th2 and Treg	Wang et al., 2016 [58]
Dendritic cells play a crucial role in the development of psoriatic inflammation, because of the production IL-12, IL-23, IL-6 [72]	WJSCs inhibit the maturation and activation of dendritic cell precursors	Kim et al., 2013 [56]
Psoriatic keratinocytes are particularly resistant to apoptosis, in psoriatic lesions over-expressed Bcl-XL, stimulated by TNF-α, is observed [73]	hWJSCs up-regulate the induction of apoptosis by attenuation of Bcl-2, Bcl-XL activation	Fong et al., 2011 [64] Han et al., 2014 [74]

WJSCs, Wharton’s Jelly Stem Cells; sHLA-G, soluble HLA-G; MSCs, Mesenchymal Stem Cells; WJ-MSCs, Wharton Jelly Mesenchymal Stem Cells; hWJSCs, human Wharton Jelly Mesenchymal Stem Cells; Bcl-XL, B-cell lymphoma-extra large.

**Table 2:** Psoriasis remission due to autologous haematopoietic stem cell transplantation.

Author	Patient	Psoriasis Course	Reason of HSCT	Myeloablative Chemotherapy	HSCT Type	Remission of Psoriasis	Comments
Adkins, 2000 [41]	K, 55 years old	Severe PS for 33 years, BSA > 60%, treated earlier with CsA, PUVA, MTX, with no improvement	CML	BU, CTX	Allo-HSCT	2 years 4 months	Post-surgery period complicated with recurring infections and acute and chronic GVHD, treated with GCS, CsA and AZA. Died on 887th day following transplant because of pneumonia and AKF
Braiteh, 2008 [76]	M, 35 years old	PS and PsA for 15 years, BSA 50%	MM	L-PAM	Auto-HSCT	>2 years follow-up	1 year of remission of MM
Mohren, 2004 [83]	M, 34 years old	PS and severe PsA for 15 years, ineffectively treated with MTX, CsA, MMF, sulfasalazine, NSAIDs and drugs in combination	PSA	CTX, L-PAM and selection of CD34+ cells from graft	PBSCT	16 months	Mild recurring PSA, with good response to MTX. Also, history of monoclonal gammopathy IgAκ, resolved months following PBSCT, no recurrence.
Mori, 2012 [75]	M, 54 years old	PS for 10 years	MDS	BU, CTX	Allo-BMT	8 months follow-up	
Woods, 2006 [43]	M, 29 years old	PS for 16 years, severe PSA for 1 year, heavily restricts performance	AA	CTX, radiotherapy	Allo-HSCT	12 months PS 5 years PsA	The 20-year follow-up after HSCT showed a recurrence of mild psoriasis limited to head skin and recurrence of PSA, well-controlled with drugs and not causing significant disability.
Held, 2012 [84]	M, 9 years old	Guttate psoriasis, erythroderma	Edwing sarcoma	BU, L-PAM	Auto-SCT (ASCR)	15 months follow-up	13 months of remission of Edwing sarcoma
Kishimoto 1997 [85]	M, 40 years old	PPP following chemotherapy (DRB, 6-MP and BH-AC), treated with local GCS and etretinate, no improvement	AML	BU, CTX	Allo-HSCT	2 years follow-up	5 months after allo-HSCT the patient developed autoimmune thyroiditis and chronic GVHD, treated with CsA and GCS for 7 months with improvement.
Rossi, 2006 [86]	M, 27 years old	PS for 2 years, treated with local GCS	Acute AA	ATG, CTX	Allo-BMT	10 years follow-up	Received short-term MTX and CsA for 314 days following BMT as a preventive measure against GVHD
Rossi, 2006 [86]	M, 50 years old	PS for 20 years (scalp, elbows)	NHL	BEAM regimen (BCNU, AC, ETO, L-PAM)	Auto-BMT	21 months	After 21 months, recurring PS restricted to elbows
Kanamori, 2002 [87]	M, 49 years old	PS for 20 years, treated with GCS externally	CML	BU, CTX, AC	Allo-BMT	2 years 6 months follow-up	Patient received short-term MTX and CsA for 150 days as a preventive measure against GVHD. After BMT, developed liver function disorder (probably related to chronic GVHD)
Slavin, 2000 [88]	M, 38 years old	Severe PS and PSA for 8 years, periodically treated with MTX and phototherapy	CML	FLU, ATG, BU	Allo-BMT NST	2 years follow-up	Patient received CsA as a preventive measure against GVHD. 32 days after BMT there was recurrence of PS, PSA and CML. CsA was discontinued with a view to inducing GVL against CML and GVA against PSA. Within a month, the patient developed macular-papular eruptions, like in GVHD, treated with GCS. Symptoms of CML, PS and PSA were resolved.

HSCT, hematopoietic stem cell transplantation; PS, psoriasis; BSA, body surface area; CsA, ciclosporin, MTX, methotrexate; PUVA, photochemotherapy UVA; CML, chronic myeloid leukemia; BU, busulfan; CTX, cyclophosmamide; GVHD, graft versus host disease; GCS, glucocorticosteroids; AZA, azatiopryne; AKF, acute kidney failure; PsA, psoriasis arthritis; MM, myeloma multiplex; L-PAM, melphalan; PBSCT, peripherial blood stem cell transplantation; MDS, myelodysplastic syndrome; MMF, mycophenolate mofetil, NSAIDs, non steroidal anti-inflammatory drugs; ATG, antithymocyte globulin; AA, aplastic anemia; ASCT, autologous stem cell transplantation; PPP, plantopalmar psoriasis pustulosa; DRB, doxorubicin; 6-MP, 6-mercaptopurine; BH-AC; AML, acute myeloid leukemia; BH-AC, behenoyl cytosine arabinoside; BCNU1,3-bis (2-chloroethyl)-1-nitroso-urea; ETO, etoposide, AC, cytarabine; CNL, chronic myeloid leukemia; FLU, flutamide; GVL, graft versus leukemia effect; GVA, graft versus autoimmunity.

**Table 3:** List of past and present clinical adipose-derived stem cell trials in psoriasis [95].

	Study	Application Method	Phase	Trial Institution and Country	NCT Number and Duration Period
1	Safety and Efficacy of UC-MSCs in Patients With Psoriasis Vulgaris	Patients will receive six UC-MSCs infusions (1 × 10^6^/kg). The first to fourth infusion will be given once a week for four weeks, then the last two infusions will be given once every two weeks.	I, II	Hospital to Academy of Military Medical Sciences, China	NCT02491658 2015–2016
2	Safety of FURESTEM-CD Inj. in Patients With Moderate to Severe Plaque-type Psoriasis	Patients will receive FURESTEM-CD (allogeneic hUCB-MSC) injection subcutaneous: 5.0 × 10^7^ cells, 1.0 × 10^7^ cells and 2.0 × 10^8^ cells for four weeks.	I	The Catholic Univ. Korea Seoul, St. Marry’s Hospital, Seoul, Republic of Korea	NCT02918123 2016–2020
